# Supplementary material for: Prostate-Specific Antigen and Testosterone Levels as Biochemical Indicators of Cognitive Function in Prostate Cancer Survivors and the Role of Diabetes
Source: J Clin Med. 2021 Nov 15;10(22):5307. doi: 10.3390/jcm10225307 (PMC8619514; doi:10.3390/jcm10225307)
Supplement: Supplementary file 1 [file jcm-10-05307-s001.zip › jcm-1405555-supplementary.pdf]

Table S1. Biochemical parameters in study group and in subgroups with and without persistently elevated PSA level

**Parameter** [Ref. value]	All n=118	Postoperative PSA <0,1 (n=97)	Postoperative PSA >0,1 (n=21)	d-Cohen	**P**
Pre-treatment PSA [ng/ml]	7,5 (5,4 – 10,7)	7,3 (5,4 – 10,0)	12,0 (5,51 – 24,6)	0,82	**0,027**
Post-surgery PSA [ng/ml]	0,006 (0,003 – 0,03)	0,003 (0,003 – 0,012)	0,157 (0,06 – 0,57)	0,41	**9,46E-20**
Current PSA [ng/ml]	0,004 (0,003 – 0,035)	0,003 (0,003 – 0,017)	0,138 (0,009 – 0,454)	0,33	**0,0003**
Free Testosterone [ng/ml]	4,54 (3,2 – 6,23)	4,57 (3,2 – 6,34)	4,4 (3,2 – 5,1)	0,34	0,28
Total Testosterone [ng/ml]	3,39 (2,57 – 4,78)	3,90 (3,10 – 5,07)	2,90 (0,13 – 4,35)	0,63	0,49
CRP [mg/l]	1,4 (0,6 – 2,7)	1,3 (0,6 – 2,8)	1,8 (1,0 – 2,7)	0,17	0,56
Wbc [G/l]	6,5 (5,78 – 7,91)	6,69 (5,77 – 7,91)	6,28 (5,83 – 7,48)	0,09	0,76

Data are shown as the median (Q25 – Q75). Inter-group differences were assessed using the Mann–Whitney U test. Effect size was measured using the Cohen’d method. PSA – prostate-specific antigen; CRP – C-reactive protein; Wbc – white blood cells. Post-treatment PSA was measured in 6 week after surgery. Significant p-values shown in bold.

Table S2. Cognitive parameters in study group and in subgroups with and without persistently elevated PSA level

**Parameter** [Ref. value]	All n=118	Postoperative PSA <0,1 (n=97)	Postoperative PSA >0,1 (n=21)	d-Cohen	**P**
SRT_C [n]	25,0 (25,0 – 25,0)	25,0 (25,0 – 25,0)	25,0 (25,0 – 25,0)	0,63	0,94
SRT_RT [ms]	282,9 234,6 – 330,7)	283,1 (248,0 – 328,1)	281,2 (228,6 – 343,9)	0,04	0,97
VM_1 [n]	5,0 (4,0 – 7,0)	5,0 (4,0 – 7,0)	6,0 (5,0 – 6,0)	0,13	0,73
VM_2 [n]	7,0 (6,0 – 8,0)	7,0 (6,0 – 8,0)	7,0 (6,0 – 8,0)	0,25	0,25
VM_3 [n]	7,0 (7,0 – 8,0)	8,0 (7,0 – 9,0)	7,0 (6,0 – 8,0)	0,41	0,07
VM_4 [n]	8,0 (7,0 – 10,0)	8,0 (7,0 – 10,0)	7,0 (6,0 – 8,0)	0,68	**0,006**
VM_5 [n]	8,0 (7,0 – 10,0)	8,0 (7,0 – 10,0)	7,0 (6,0 – 8,0)	0,64	**0,008**
VMDT_C [n]	6,0 (5,0 – 7,0)	6,0 (5,0 – 7,0)	5,0 (5,0 – 7,0)	0,75	0,47
GoNoGo_C [n]	74,0 (73,0 – 75,0)	74,0 (72,0 – 75,0)	74,0 (73,0 – 75,0)	0,12	0,83
GoNoGo_RT [ms]	365,8 (321,5 – 415,9)	366,5 (321,6 – 417,0)	362,5 (313,1 – 415,2)	0,28	0,34
GoNoGo IncGO [n]	1,0 (0,0 – 2,0)	1,0 (0,0 – 2,0)	1,0 (0,0 – 2,0)	0,26	0,90
GoNoGo IncNoGo [n]	5,0 (2,0 – 8,0)	5,0 (2,0 – 8,0)	6,0 (3,0 – 8,0)	0,12	0,48
VWMT_C [n]	5,0 (3,0 – 6,0)	5,0 (4,0 – 7,0)	4,0 (3,0 – 6,0)	0,19	0,33
VWMT_CRT [ms]	3138,5 (2541,0 – 4429,0)	3015,0 (2421,0 – 4234,0)	3843,0 (2828,0 – 5414,0)	0,34	0,058
VWMT_IRT [ms]	3563,5 (0,0 – 5117,0)	3625,0 (0,0 – 5355,0)	2953,0 (0,0 – 4422,0)	0,06	0,72

Data are shown as the median (Q25 – Q75) or number (%). Inter-group differences were assessed using the Mann–Whitney U test. Effect size was measured using the Cohen d method; SRT_C – simple reaction time test (number of correct answers); SRT_RT – simple reaction time test (average reaction time); VM_1 – VM_5 – verbal memory (number of words remembered in each of the five attempts); VMDT – verbal memory deferred test (number of words remembered); GoNoGo_C – GoNoGo test (number of correct answers); RT – reaction time; IncGo – number of incorrect Go answers; IncNoGo – incorrect NoGo answers; VWMT_C – visuospatial working memory task (number of correct answers); CRT – average response time for correct answers; IRT – average response time for incorrect answers. Post-treatment PSA was measured in 6 week after surgery. Significant p-values shown in bold.

Table S3. R-Spearman correlations of cognitive test results and free/total testosterone level

Parameter	Total testosterone	p	Free testosterone	p
**SRT_C**	*-0,005489*	*ns.*	0,010411	ns.
**SRT_RT**	-0,173650	0,059	-0,175864	ns.
**VM_1**	-0,115720	ns.	-0,114129	ns.
**VM_2**	-0,080680	ns.	-0,107193	ns.
**VM_3**	0,007652	ns.	-0,001282	ns.
**VM_4**	0,058022	ns.	0,065259	ns.
**VM_5**	0,064911	ns.	0,073037	ns.
**VMDT_C**	0,014506	ns.	0,059360	ns.
**GoNoGo_C**	0,007978	ns.	0,103684	ns.
**GoNoGo_RT**	-0,051953	ns.	-0,068426	ns.
**GoNoGo IncGO**	-0,038768	ns.	-0,102018	ns.
**GoNoGo IncNoGo**	-0,051987	ns.	-0,074934	ns.
**VWMT_C**	-0,023409	ns.	-0,047978	ns.
**VWMT_CRT**	0,049630	ns.	-0,001287	ns.
**VWMT_IRT**	0,022038	ns.	0,052042	ns.

SRT_C – simple reaction time test (number of correct answers); SRT_RT – simple reaction time test (average reaction time); VM_1 – VM_5 – verbal memory (number of words remembered in each of the five attempts); VMDT – verbal memory deferred test (number of words remembered); GoNoGo_C – GoNoGo test (number of correct answers); RT – reaction time; IncGo – number of incorrect Go answers; IncNoGo – incorrect NoGo answers; VWMT_C – visuospatial working memory task (number of correct answers); CRT – average response time for correct answers; IRT – average response time for incorrect answers. Significant p-values shown in bold.

Table S4. R-Spearman correlations of cognitive test results and free testosterone level in subgroups with and without diabetes

Parameter	Free testosterone in NONdiabetes (n=97)	p	Free testosterone in Diabetes (n=19)	P
SRT_C	0,012882	ns.	0,087565	ns.
SRT_RT	-0,188325	0,07	0,057772	ns.
VM_1	-0,141837	ns.	0,219458	ns.
VM_2	-0,175111	0,08	0,346977	ns.
VM_3	-0,068813	ns.	0,389517	0,09
VM_4	0,052876	ns.	0,169734	ns.
VM_5	0,062735	ns.	0,189547	ns.
VMDT_C	-0,061760	ns.	0,395459	*0,09*
GoNoGo_C	0,005478	ns.	0,689345	**0,001**
GoNoGo_RT	-0,111770	ns.	0,052270	ns.
GoNoGo IncGO	-0,003377	ns.	-0,627646	**0,004**
GoNoGo IncNoGo	-0,037703	ns.	-0,178284	ns.
VWMT_C	0,061760	ns.	0,280092	ns.
VWMT_CRT	-0,093871	ns.	-0,052270	ns.
VWMT_IRT	0,052681	ns.	-0,289367	ns.

SRT_C – simple reaction time test (number of correct answers); SRT_RT – simple reaction time test (average reaction time); VM_1 – VM_5 – verbal memory (number of words remembered in each of the five attempts); VMDT – verbal memory deferred test (number of words remembered); GoNoGo_C – GoNoGo test (number of correct answers); RT – reaction time; IncGo – number of incorrect Go answers; IncNoGo – incorrect NoGo answers; VWMT_C – visuospatial working memory task (number of correct answers); CRT – average response time for correct answers; IRT – average response time for incorrect answers. PSA – prostate-specific antigen. Significant p-values shown in bold.

Table S5. Demographic and clinical data in Non-diabetes and Diabetes group

Parameter		Non-diabetes (n=99)	Diabetes (n=19)	d-Cohen	P
**Age (y)**		65,0 (59,0 – 70,0)	70,0 (65,0 – 73,0)	0,52	0,04
**BMI (kg/m^2)**		27,2 (25,3 – 29,7)	27,4 (25,8 – 29,7)	0,16	0,67
**Months from surgery**		20,0 (10,0 – 36)	16,0 (6,0 – 24,0)	0,58	0,10
**Hypertension (n, %)**		51 (51,5%)	14 (73,5%)		0,077
**MI (n, %)**		8 (8%)	2 (10,5%)		0,73
**Stroke (n, %)**		7 (7%)	0 (0%)		0,23
**Education**	**Basic (n, %)**	6 (6%)	0 (0%)		0,92
	**Vocational (n, %)**	28 (28%)	5 (26,5%)		
	**Secondary (n, %)**	27 (27%)	9 (47%)		
	**Higher (n, %)**	38 (38,5%)	5 (26,5%)		
**Physical activity**	**None (n, %)**	30 (30%)	10 (52,5%)		**0,01**
	**< 1x/week (n, %)**	20 (20%)	4 (21%)		
	**<3x/week**	49 (50%)	5 (26,5%)		
**GRADE**	**1 (n, %)**	58 (58,5%)	12 (63%)		0,88
	**2 (n, %)**	32 (32%)	3 (16%)		
	**3 (n, %)**	5 (5%)	0 (0%)		
	**4 (n, %)**	2 (2%)	2 (10,5%)		
	**5 (n, %)**	2 (2%)	2 (10,5%)		
**Nicotinism (n, %)**		46 (46,5%)	7 (37%)		0,44
Pre-treatment PSA [ng/ml]		7,4 (5,4 – 10,7)	8,0 (5,8 – 12,0)	0,10	0,37
Post-surgery PSA [ng/ml]		0,008 (0,003 – 0,03)	0,003 (0,002 – 0,05)	0,22	0,45
Current PSA [ng/ml]		0,003 (0,003 – 0,035)	0,006 (0,003 – 0,098)	3,7	0,54
Free Testosterone [ng/ml]		4,7 (3,49 – 6,32)	3,85 (2,52 – 5,3)	0,38	0,10
Total Testosterone [ng/ml]		4,24 (3,48 – 5,44)	3,04 (1,50 – 3,26)	1,10	**0,02**
CRP [mg/l]		1,4 (0,6 – 2,7)	1,7 (1,1 – 3,9)	0,37	0,12
Wbc [G/l]		6,55 (5,77 – 7,78)	6,33 (5,92 – 7,96)	0,13	0,76

Data are shown as the median (Q25 – Q75) or number (%). Inter-group differences were assessed using the Mann–Whitney U test. Effect size was measured using the Cohen d method. BMI- body mass index; MI – myocardial infarction; GRADE: group of grading system classification; y – years; m – months. PSA – prostate-specific antigen; CRP – C-reactive protein; Wbc – white blood cells. Post-treatment PSA was measured in 6 week after surgery. Significant p-values shown in bold.

Table S6. Cognitive parameters in non-diabetes and diabetes group

**Parameter** [Ref. value]	Non-diabetes (n=99)	Diabetes (n=19)	d-Cohen	**P**
SRT_C	25,0 (25,0 – 25,0)	25,0 (25,0 – 25,0)	0,63	0,94
SRT_RT	276,7 (231,5 – 325,2)	307,7 (271,0 – 358,4)	0,33	**0,047**
VM_1	5,0 (4,0 – 7,0)	6,0 (5,0 – 6,0)	0,06	0,91
VM_2	7,0 (6,0 – 8,0)	7,0 (6,0 – 8,0)	0,25	0,38
VM_3	7,0 (7,0 – 9,0)	7,0 (6,0 – 8,0)	0,33	0,20
VM_4	8,0 (7,0 – 10,0)	8,0 (6,0 – 8,0)	0,45	0,077
VM_5	8,0 (7,0 – 10,0)	8,0 (6,0 – 8,0)	0,41	0,09
VMDT_C	6,0 (5,0 – 7,0)	6,0 (4,0 – 7,0)	0,17	0,70
GoNoGo_C	74,0 (73,0 – 75,0)	74,0 (69,0 – 75,0)	0,20	0,91
GoNoGo_RT	366,5 (321,5 – 422,1)	364,3 (313,6 – 389,2)	0,49	0,13
GoNoGo IncGO	1,0 (0,0 – 2,0)	1,0 (0,0 – 6,0)	0,20	0,63
GoNoGo IncNoGo	4,0 (2,0 – 7,0)	8,0 (6,0 – 9,0)	0,75	**0,001**
VWMT_C	6,0 (5,0 – 7,0)	6,0 (3,0 – 7,0)	0,30	0,99
VWMT_CRT	3144,0 (2541,0 – 4429,0)	3015,0 (2440,0 – 4586,0)	0,13	0,82
VWMT_IRT	3484,0 (0,0 – 5117,0)	3650,0 (0,0 – 5469,0)	0,01	0,98

Data are shown as the median (Q25 – Q75) or number (%). Inter-group differences were assessed using the Mann–Whitney U test. Effect size was measured using the Cohen d method; SRT_C – simple reaction time test (number of correct answers); SRT_RT – simple reaction time test (average reaction time); VM_1 – VM_5 – verbal memory (number of words remembered in each of the five attempts); VMDT – verbal memory deferred test (number of words remembered); GoNoGo_C – GoNoGo test (number of correct answers); RT – reaction time; IncGo – number of incorrect Go answers; IncNoGo – incorrect NoGo answers; VWMT_C – visuospatial working memory task (number of correct answers); CRT – average response time for correct answers; IRT – average response time for incorrect answers. Significant p-values shown in bold

Table S7. Multiple regression model coefficients on cognitive test results

	**SRT_RT**						
	**β**	**S.E. of β**	**b**	**S.E. of b**	**t**	**p**	**95%C.I. Low/ Up**
Intercept			**219,3**	**83,9**	**2,61**	**0,01**	**52,9/385,6**
Age	0,14	0,10	1,78	1,25	1,41	0,15	-0,7/4,27
Duration from surgery	-0,07	0,09	-0,28	0,37	-0,76	0,44	-1,0/0,45
Pre-treatment PSA	-0,02	0,10	-0,27	1,24	-0,21	0,82	-2,7/2,19
Post-surgery PSA	0,06	0,10	3,4	5,7	0,59	0,55	-8,0/14,8
Current PSA	**0,22**	**0,10**	**1,94**	**0,92**	**2,11**	**0,03**	**0,11/3,7**
Total testosterone	-0,08	0,09	-2,8	3,2	-0,86	0,38	-9,31/3,6
Diabetes	0,01	0,09	3,7	21,8	0,16	0,86	-39,6/47,0
GRADE	-0,09	0,12	-7,9	10,1	-0,78	0,43	-28,0/12,1
	**VM_1**						
	**β**	**S.E. of β**	**b**	**S.E. of b**	**t**	**p**	**95%C.I. Low/ Up**
Intercept			**10,0**	**1,56**	**6,42**	**<0,0001**	**6,97/13,1**
Age	**-0,27**	**0,09**	**-0,06**	**0,02**	**-2,87**	**0,004**	**-0,11/-0,02**
Duration from surgery	-0,2	0,09	-0,001	0,006	-0,23	0,81	-0,01/0,01
Pre-treatment PSA	0,08	0,10	0,01	0,02	0,85	0,39	-0,02/0,06
Post-surgery PSA	**-0,2**	**0,10**	**-0,21**	**0,10**	**-2,04**	**0,04**	**-0,43/-0,006**
Current PSA	**-0,23**	**0,10**	**-0,03**	**0,01**	**-2,29**	**0,02**	**-0,07/-0,005**
Total testosterone	-0,16	0,09	-0,10	0,06	-1,74	0,08	-0,22/0,014
Diabetes	0,09	0,09	0,39	0,40	0,96	0,33	-0,41/1,2
GRADE	0,07	0,11	0,11	0,18	0,61	0,53	-0,25/0,49
	**VM_2**						
	**β**	**S.E. of β**	**b**	**S.E. of b**	**t**	**p**	**95%C.I. Low/ Up**
Intercept			**9,0**	**1,72**	**5,23**	**<0,0001**	**5,6/12,4**
Age	-0,08	0,10	-0,02	0,02	-0,81	0,41	-0,07/0,03
Duration from surgery	-0,09	0,09	-0,007	0,007	-0,91	0,36	-0,02/0,008
Pre-treatment PSA	0,05	0,10	0,01	0,02	0,50	0,61	-0,03/0,06
Post-surgery PSA	-0,07	0,10	-0,08	0,11	-0,68	0,49	-0,31/0,15
Current PSA	**-0,22**	**0,10**	**-0,03**	**0,01**	**-2,1**	**0,03**	**-0,077/-0,002**
Total testosterone	-0,14	0,09	-0,1	0,06	-1,49	0,13	-0,23/0,03
Diabetes	-0,03	0,09	-0,17	0,44	-0,39	0,69	-1,06/0,71
GRADE	0,02	0,12	0,03	0,2	0,16	0,86	-0,37/0,44
	**VM_4**						
	**β**	**S.E. of β**	**b**	**S.E. of b**	**t**	**p**	**95%C.I. Low/ Up**
Intercept			**13,2**	**1,81**	**7,2**	**<0,0001**	**9,64/16,8**
Age	**-0,26**	**0,09**	**-0,07**	**0,02**	**-2,65**	**0,009**	**-0,12/-0,01**
Duration from surgery	0,03	0,09	0,003	0,008	0,39	0,69	-0,01/0,01
Pre-treatment PSA	-0,08	0,10	-0,02	0,02	-0,85	0,39	-0,07/0,03
Post-surgery PSA	0,11	0,10	0,13	0,12	1,11	0,26	-0,10/0,38
Current PSA	**-0,22**	**0,10**	**-0,04**	**0,01**	**-2,21**	**0,028**	**-0,08/-0,004**
Total testosterone	-0,08	0,09	-0,06	0,07	-0,9	0,35	-0,2/0,07
Diabetes	-0,05	0,09	-0,25	0,47	-0,53	0,59	-1,19/0,68
GRADE	-0,03	0,11	-0,07	0,219	-0,33	0,73	-0,05/0,36
	**VM_5**						
	**β**	**S.E. of β**	**b**	**S.E. of b**	**t**	**p**	**95%C.I. Low/ Up**
Intercept			**12,8**	**1,8**	**7,07**	**<0,0001**	**9,26/16,4**
Age	**-0,24**	**0,09**	**-0,06**	**0,02**	**-2,52**	**0,013**	**-0,12/-0,014**
Duration from surgery	0,03	0,09	0,002	0,008	0,36	0,71	-0,01/0,02

Pre-treatment PSA	-0,07	0,10	-0,02	0,02	-0,77	0,44	-0,07/0,03
Post-surgery PSA	0,10	0,10	0,13	0,12	1,04	0,29	-0,11/0,37
Current PSA	**-0,23**	**0,10**	**-0,04**	**0,019**	**-2,32**	**0,02**	**-0,08/-0,006**
Total testosterone	-0,07	0,09	-0,05	0,07	-0,77	0,43	-0,19/0,08
Diabetes	-0,03	0,09	-0,16	0,47	-0,35	0,72	-1,1/0,77
GRADE	-0,01	0,11	-0,01	0,21	-0,08	0,93	-0,45/0,41
	**VMDT_C**						
	**β**	**S.E. of β**	**b**	**S.E. of b**	**t**	**p**	**95%C.I. Low/ Up**
Intercept			**10,9**	**1,9**	**5,7**	**<0,0001**	**7,18/14,7**
Age	**-0,27**	**0,10**	**-0,07**	**0,02**	**-2,7**	**0,006**	**-0,13/-0,02**
Duration from surgery	0,06	0,09	0,005	0,008	0,66	0,50	-0,11/0,05
Pre-treatment PSA	-0,04	0,10	-0,01	0,028	-0,46	0,64	-0,06/0,04
Post-surgery PSA	-0,04	0,10	-0,05	0,13	-0,39	0,69	-0,31/0,20
Current PSA	*-0,18*	*0,10*	*-0,03*	*0,02*	*-1,77*	*0,07*	*-0,07/0,004*
Total testosterone	-0,03	0,09	-0,02	0,07	-0,35	0,72	-0,17/0,12
Diabetes	0,04	0,09	0,25	0,49	0,50	0,61	-0,73/1,23
GRADE	0,03	0,11	0,07	0,23	0,3	0,75	-0,38/0,52
	**GoNoGo_RT**						
	**β**	**S.E. of β**	**b**	**S.E. of b**	**t**	**p**	**95%C.I. Low/ Up**
Intercept			**192,3**	**59,9**	**3,21**	**0,001**	**735/311,0**
Age	**0,32**	**0,09**	**3,01**	**0,89**	**3,35**	**0,001**	**1,23/4,79**
Duration from surgery	-0,07	0,09	-0,21	0,26	-0,80	0,42	-0,74/0,31
Pre-treatment PSA	0,04	0,10	0,37	0,88	0,41	0,67	-1,38/2,12
Post-surgery PSA	-0,07	0,10	-3,01	4,11	-0,73	0,46	-11,1/5,13
Current PSA	-0,11	0,10	-0,76	0,65	-1,16	0,24	-2,06/0,53
Total testosterone	-0,05	0,09	-1,42	2,33	-0,61	0,54	-6,06/3,2
Diabetes	**-0,21**	**0,09**	**-34,8**	**15,6**	**-2,23**	**0,02**	**-65,7/-3,89**
GRADE	-0,001	0,11	-0,11	7,24	-0,01	0,98	-14,4/14,2
	**GoNoGo_IncNoGo**						
	**β**	**S.E. of β**	**b**	**S.E. of b**	**t**	**p**	**95%C.I. Low/ Up**
Intercept			0,71	3,66	0,19	0,84	-6,5/7,97
Age	0,05	0,10	0,03	0,05	0,55	0,57	-0,07/0,13
Duration from surgery	0,14	0,09	0,02	0,016	1,43	0,15	-0,008/0,05
Pre-treatment PSA	0,03	0,10	0,02	0,05	0,37	0,70	-0,08/0,12
Post-surgery PSA	-0,08	0,10	-0,2	0,25	-0,8	0,42	-0,70/0,29
Current PSA	0,01	0,10	0,007	0,04	0,18	0,85	-0,07/0,08
Total testosterone	0,08	0,09	0,12	0,14	0,90	0,36	-0,15/0,41
Diabetes	**0,23**	**0,09**	**2,25**	**0,95**	**2,36**	**0,02**	**0,36/4,14**
GRADE	0,16	0,11	0,60	0,44	1,36	0,17	-0,27/1,48
	**VWMT_CRT**						
	**β**	**S.E. of β**	**b**	**S.E. of b**	**t**	**p**	**95%C.I. Low/ Up**
Intercept			-237,6	1680,1	-0,14	0,88	-3568/3092
Age	**0,20**	**0,10**	**50,4**	**25,1**	**2,0**	**0,04**	**0,58/100,3**
Duration from surgery	0,06	0,09	4,9	7,4	0,65	0,51	-9,9/19,7
Pre-treatment PSA	-0,02	0,10	-4,7	24,8	-0,19	0,84	-54,1/44,5
Post-surgery PSA	-0,02	0,10	-31,1	115,3	-0,27	0,78	-269/197
Current PSA	**0,20**	**0,10**	**35,9**	**18,4**	**1,94**	**0,04**	**-0,64/72,4**
Total testosterone	0,10	0,09	72,1	65,5	1,09	0,27	-57,8/2,2
Diabetes	-0,01	0,09	-61,4	437,9	-0,14	0,88	-929/806
GRADE	0,03	0,12	51,4	203,2	0,25	0,80	-351/454
